# Uncertainty in Population Estimates for Endangered Animals and Improving the Recovery Process

**DOI:** 10.3390/ani3030745

**Published:** 2013-08-13

**Authors:** Aaron M. Haines, Matthew Zak, Katie Hammond, J. Michael Scott, Dale D. Goble, Janet L. Rachlow

**Affiliations:** 1 Applied Conservation Lab, Department of Biology, Millersville University, Millersville, PA 17551, USA; E-Mail: matthewmzak@gmail.com; 2 Ecology and Conservation Biology Program, College of Natural Resources, University of Idaho, Moscow, ID 83844, USA; E-Mail: brit0213@vandals.uidaho.edu; 3 Department of Fish and Wildlife Resources, College of Natural Resources, University of Idaho, Room 103, Moscow, ID 83844, USA; E-Mails: mscott@uidaho.edu (J.M.S.); jrachlow@uidaho.edu (J.L.R.); 4 College of Law, University of Idaho, Law 201, Moscow, ID 83844, USA; E-Mail: gobled@uidaho.edu

**Keywords:** delisting, Endangered Species Act, minimum detectable difference, population size, recovery criteria, variance

## Abstract

**Simple Summary:**

The objective of our study was to evaluate the mention of uncertainty (*i.e*., variance) associated with population size estimates within U.S. recovery plans for endangered animals. To do this we reviewed all finalized recovery plans for listed terrestrial vertebrate species. We found that more recent recovery plans reported more estimates of population size and uncertainty. Also, bird and mammal recovery plans reported more estimates of population size and uncertainty. We recommend that updated recovery plans combine uncertainty of population size estimates with a minimum detectable difference to aid in successful recovery.

**Abstract:**

United States recovery plans contain biological information for a species listed under the Endangered Species Act and specify recovery criteria to provide basis for species recovery. The objective of our study was to evaluate whether recovery plans provide uncertainty (e.g., variance) with estimates of population size. We reviewed all finalized recovery plans for listed terrestrial vertebrate species to record the following data: (1) if a current population size was given, (2) if a measure of uncertainty or variance was associated with current estimates of population size and (3) if population size was stipulated for recovery. We found that 59% of completed recovery plans specified a current population size, 14.5% specified a variance for the current population size estimate and 43% specified population size as a recovery criterion. More recent recovery plans reported more estimates of current population size, uncertainty and population size as a recovery criterion. Also, bird and mammal recovery plans reported more estimates of population size and uncertainty compared to reptiles and amphibians. We suggest the use of calculating minimum detectable differences to improve confidence when delisting endangered animals and we identified incentives for individuals to get involved in recovery planning to improve access to quantitative data.

## 1. Introduction

In the United States, wildlife species of conservation concern are listed as either “endangered” or “threatened” depending on their status and probability of extinction as outlined within the Endangered Species Act [1]. The Endangered Species Act of 1973 [1] was created to provide a means by which endangered and threatened species and the ecosystems upon which they depend could be conserved (ESA sec. 2(5b)). The term “endangered” refers to “any species that is in danger of extinction throughout all or a significant portion of its range,” and the term “threatened” refers to “any species which is likely to become an endangered species within the foreseeable future throughout all or a significant portion of its range” [1]. Each species placed under the ESA is given a recovery plan. The U.S. Fish and Wildlife Service [2] bases recovery of listed species on proposed recovery criteria stipulated within approved recovery plans. Recovery plans contain all the available biological information for a listed species and specify the recovery criteria (a.k.a., recovery goals, recovery benchmarks, or recovery objectives) that (when achieved) will provide the basis for downlisting (*i.e.*, reclassifying a species from endangered to threatened status) or delisting (*i.e.*, removing a species from the protection of the ESA) [2]. 

Since its enactment, 1,476 species have been listed under the ESA, with 29 species delisted as recovered [3]. A total of 1,142 listed species have recovery plans to guide recovery efforts [3]. In 1988, an amendment required that all recovery plans include “objective, measurable” delisting criteria [4]. Population size is an example of such a criteria. Setting population targets is important because the targets allow biologists to determine if conservation efforts are successful, how much additional protection is needed, how much harvest can be allowed and which threats may need to be addressed first to increase population levels [5]. Gerber and Hatch [4] found that listed species with more quantitative recovery criteria (e.g., population size) had improving recovery status. Thus, recovery goals for listed species help play a central role in applying science to policy and translating policy into action [6]. 

Tear *et al.* [7,8] suggested that quantitative recovery criteria do not necessarily promote increasing populations. Reasons for this disconnect may include political pressure for lower recovery criteria [9], or a “shifting baseline syndrome” [10] in which successive generations of wildlife managers use baseline conditions they experienced during their careers rather than the past, thus resulting in lower expectations for recovery objectives [6]. The question remains on how best to deal with recovery criteria that have already been stipulated? Tear *et al.* [6,8] suggested that population size estimates should incorporate a measure of a margin of error to create more robust estimates for stipulated recovery criteria with an evaluation of error and uncertainty associated with quantitative criteria. 

The objective of our study was to evaluate whether recovery plans calculate uncertainty for quantitative data by using population size for listed terrestrial vertebrate species as an example. We realize the complexity of the recovery process goes well beyond the issue of increasing species population size, but our goal was to focus on improving the recovery process when population size was used as a recovery criterion. The use of uncertainty calculated for species’ population size estimates may help prevent species from being downlisted or delisted prematurely. Our specific objectives included: (1) identify percentage of recovery plans that stipulate a current population size, (2) if a measure of uncertainty was associated with species estimates of current population size (3) identify the number of recovery plans that stipulate a population size as a recovery criteria, and (4) provide recommendations to help incorporate error or uncertainty into the recovery process.

## 2. Experimental Section

We reviewed all finalized recovery plans for species listed as threatened or endangered by the ESA in the United States (U.S.) through April of 2013. Digital copies of the plans were obtained as Portable Document Files (PDFs) from the web-based USFWS Threatened and Endangered Species System (TESS) [3]. In our analysis we focused on listed terrestrial vertebrate species (*i.e.*, Class Amphibia, Aves, Mammalia and Reptilia). 

Key word searches were conducted within each PDF in order to locate the following information: population, downlist, delist, deviation, error, variance, variability, mean, mode, median, S.D., standard, confidence, interval, maximum, and minimum. Read-only recovery documents were manually searched to find these key words. 

For each recovery plan, we tried to find population estimates and associated variance for each species. However, variations and limitations within recovery plans can lead to misinterpretations, thus we adopted the following conventions to standardize language used within the plans: (1) If the range of a listed species extended beyond the borders of the U.S., we included only the population data and variance for the U.S. range. (2) We did not include captive populations in determining the total number of individuals in a population size. (3) When recovery criteria specified that population sizes should be stabilized and/or maintained, we defined these recovery criteria as the population size at the time the recovery plan was drafted. 

A chi-square analysis was used to determine if there were any significant differences in the number of recovery plans that provided a current population size estimate, a calculated uncertainty in the current population estimate and a population size as a recovery criterion between each decade (*i.e.*, 1980–1989, 1990–1999, 2000–2009, 2010–2013) and between each Class of terrestrial vertebrate (*i.e.*, Amphibia, Aves, Mammalia and Reptilia). Statistical significance was based on a *p*-value < 0.05 and all statistical tests were conducted using Minitab^®^ [11]. 

## 3. Results and Discussion

### 3.1. By the Numbers

A total of 200 listed terrestrial vertebrate species out of 240 had completed recovery plans [3]. We found 59% of completed recovery plans specifying a current population size and 14.5% specifying a variance for the population size estimate. Of the recovery plans that stipulated a population size as downlisting criteria, 24% reported variance for the current population size. Of the recovery plans that stipulated a population size for delisting criteria, 20% reported a variance for the current population size. 

**Figure 1:**
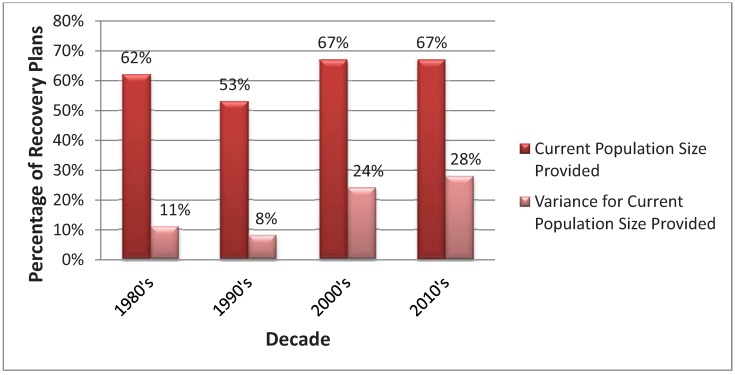
Comparison in the percentage of terrestrial vertebrate recovery plans that provide a current population and provide an estimate of uncertainty (e.g., variance) for the current population size estimate by decade.

**Figure 2:**
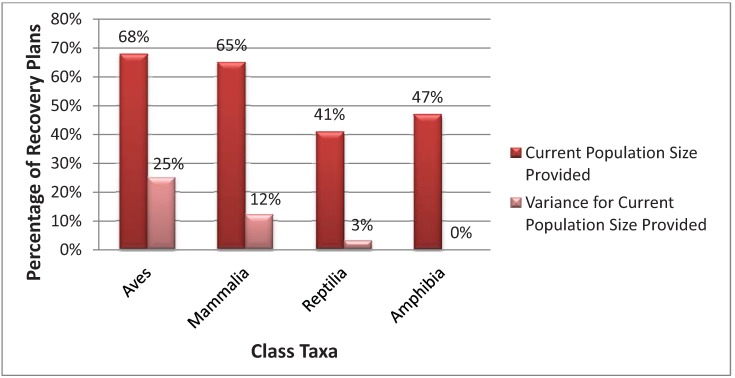
Comparison in the percentage of terrestrial vertebrate recovery plans that provide a current population and provide an estimate of uncertainty (e.g., variance) for the current population size estimate by animal Class taxa.

The percentage of recovery plans that provide a current population size did not differ significantly by decade (χ^2^ = 2.10, *p*-value = 0.55), but the percentage of recovery plans that provided uncertainty or an estimate of variance for the current population size did (χ^2^ = 16.04, *p*-value = 0.001) with the 2000s and 2010s reporting more variance (Figure 1). The percentage of recovery plans that provide a current population size was significantly different between terrestrial vertebrate Classes (χ^2^ = 9.57, *p*-value = 0.02), and the percentage of recovery plans that provided uncertainty or an estimate of variance for the current population size also differed between animal Classes (χ^2^ = 37.80, *p*-value < 0.01) (Figure 2). More recovery plans for the taxa Class Aves and Mammalia reported more estimates of current population size and variance for population size in comparison to Reptilia and Amphibia (Figure 2). 

We found that 43% of the recovery plans stipulated a population size as a recovery criterion. The percentage of avian recovery plans (59%) that stipulated a population size as a recovery criterion was significantly higher compared to reptiles (38%), mammals (31%) and amphibians (24%) (χ^2^ = 18.05, *p*-value < 0.01). Also, the percentage of the most recent recovery plans written in the 2010s (61%) that stipulated a population size as a recovery criterion was higher compared to the 2000s (41%), 1990s (42%) and 1980s (40%), however this difference was not significant (χ^2^ = 6.57, *p*-value *=* 0.08). 

Gerber & Hatch [4] found that recovery plans for endangered and threatened species have improved since 1998 because recovery criteria and quantitative data are more frequently included in recovery documents, although they found that the number of quantitative recovery criteria did not increase significantly for animals. We found that since the 1990s the number of recovery plans for terrestrial vertebrates have improved by incorporating more uncertainty into their estimate of population size (Figure 1). Also, in comparison to Gerber & Hatch [4], we found a higher percentage of recent recovery plans (2010s) for animals have included more quantitative recovery criteria (*i.e.*, population size), albeit not significantly. 

### 3.2. Improving Recovery

Overall, the ESA has been making a positive difference for species at risk of extinction [12] and we identified improvements in the reporting of quantitative data in recovery plans for terrestrial vertebrates. However, less than a quarter of listed terrestrial vertebrates (23%) recovery plans reported variance or uncertainty with their population size estimates. This may be the result of data limitations and inadequate resources for robust survey efforts [12]. Tear *et al.* [8] recommended that population estimates “should use a central tendency (mean, median, and mode) and/or the upper confidence interval to incorporate a greater margin of error and create more robust estimates for stipulated recovery criteria.” This requires the need for more rigorous survey efforts that incorporate a measure of uncertainty. For example, the recovery plan for the Marbled Murrelet (*Brachyramphus marmoratus*) recommended that the development of more robust consistent survey efforts be conducted before a current population size is defined [13]. 

Tear *et al.* [6,8] suggested that population size estimates should incorporate error or uncertainty when stipulating recovery criteria so species are not delisted prematurely. One approach we recommend for developing more detailed and robust recovery criteria when defining population size is by incorporating uncertainty into calculations of a minimum detectable difference between current population size and specified recovery criteria for delisting [14]. A minimum detectable difference (MDD) represents the smallest difference or change that would be statistically significant when comparing different samples depending on the variance of the samples and a defined level of uncertainty [14]. The equation below can be used to determine MDD (indicated with σ):

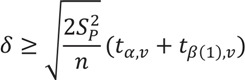


In this equation, n is the specified sample size (*i.e.*, number of surveys used to estimate population size), s^2^_p_ is the variance in the population size estimates, and *t_α,v_* and *t_β(1),v_* are applied by using a one-tailed t-value at a significance level of say 0.10. The calculation of a MDD can then be modified to determine how much larger a recovery criteria for population size must be to ensure 90% confidence for recovery. If delisting were to be considered for a listed species, a biologist could calculate MDD, based on past survey data, and then add the MDD to the species’ recovery criteria. If the current population size is greater than the sum of MDD and the species’ recovery criteria then the biologist would be >90% confident the species had achieved the recovery criteria. This could provide biologists more confidence to downlist or delist a species based on best available science, rather than strong political pressure. 

For example, in order for the Whooping crane (*Grus Americana*) to be considered for downlisting, one of the objectives is for three self-sustaining populations of cranes, of relatively similar size, to total 360 birds over 10 years [15]. Based on the Whooping crane recovery plan [15], the total annual population size in the last 10 years have been 219, 248, 244, 255, 256, 270, 292, 317, 335 and 343. This gives us an n = 10, s^2^_p_ = 1,756, a 2-tailed value = 1.83 and a one-tailed value = 1.37 for 90% confidence at nine degrees of freedom. This produces an MDD of 46.98 or 47. Thus, a crane biologist would be >90% confident the recovery criteria was achieved when the total crane population size was 407. However, this calculation would have to be revisited as the Whooping crane populations got closer to the actual recovery criteria. 

In order to calculate a MDD for endangered species, robust survey efforts need to be either developed or re-visited for each species. Unfortunately for many species, there is a lack of resources and funding available to government agencies to obtain these data [12]. Many have recommended that species recovery be part of a dynamic adaptive management process where field biologists and other government personnel work more with academics and other research scientists to pool resources and obtain required quantitative data [4,12,16,17,18]. This would be especially true for reptiles and amphibians.

However, academics may have no incentive to help with the recovery process because faculty promotion may be based on attaining grant money and authoring numerous publications rather than being part of a recovery team. This may be the case for some large research based institutions, however there are numerous benefits academic faculty can take advantage of by being part of a recovery team. As well as professional development, faculty promotion can also be based on excellence in teaching and community service. Being part of a recovery team is an excellent form of community outreach and service, especially if faculty help lead local citizen science groups. For teaching excellence, faculty can get students involved in the recovery process as an applied teaching platform that involves active learning, service learning, project based learning and cooperative learning within their courses including opportunities for student internships and research. This type of hands on student learning can lead to better student evaluation scores [19], which can also be used in the faculty promotion process. Thus, if implemented properly, recovery planning can benefit academic faculty members. 

Other recovery team members that are focused and represent diverse entities such as federal/state/local governments, non-government conservation organizations, local stakeholders, *etc.*, have also been shown to improve the recovery plan process [12,16,17]. Non-academic based incentives that can help maintain recovery team diversity may include opportunities to secure congressional funding for activities involved in restoration programs, the mobilization of resources from different entities to apply for matching grants, technical assistance on stewardship activities, being part of a recovery process or a success story for marketing purposes, and/or the development of a government review process that rewards outreach and collaborative behaviors through awards and thank you letters [20].

## 4. Conclusions

Few recovery plans report a measure of uncertainty or variance for their population size estimates. A measure of variance can be used in calculating a MDD to prevent premature delisting of a species. One could argue that calculating a MDD to ensure confidence in the delisting process may be overly cautious and would unnecessarily prolong species recovery. However, Doremus and Pagel [21] stated that delisting should be a cautious undertaken because the legislative and judicial history of the ESA is based on ‘institutional caution’ [22] and the negative consequences of erroneous delisting (*i.e.*, extinction) outweigh the costs of retaining species on the list. In addition, Neel *et al.* [23] found that recovery criteria specified in recovery plans for listed species was too low to ensure their long-term persistence. They also recommended that more species biology understanding and a clearer articulation of logic be used when choosing abundances for species recovery. We believe the calculation of a MDD can help address some of these concerns. Also, when government personnel pool resources with academics to incorporate uncertainty into species recovery criteria this may prevent premature species delisting and the need for possible re-listing, which may reduce overall costs in the recovery process. 
